# Cost-Effectiveness of Rapid Syphilis Screening in Prenatal HIV Testing Programs in Haiti

**DOI:** 10.1371/journal.pmed.0040183

**Published:** 2007-05-29

**Authors:** Bruce R Schackman, Christopher P Neukermans, Sandy N. Nerette Fontain, Claudine Nolte, Patrice Joseph, Jean W Pape, Daniel W Fitzgerald

**Affiliations:** 1 Department of Public Health, Weill Medical College, Cornell University, New York, New York, United States of America; 2 Groupe Haitien d'Etude du Sarcome de Kaposi et des Infections Opportunistes, Port-au-Prince, Haiti; 3 Department of Medicine, Weill Medical College, Cornell University, New York, New York, United States of America; Harvard School of Public Health, United States of America

## Abstract

**Background:**

New rapid syphilis tests permit simple and immediate diagnosis and treatment at a single clinic visit. We compared the cost-effectiveness, projected health outcomes, and annual cost of screening pregnant women using a rapid syphilis test as part of scaled-up prenatal testing to prevent mother-to-child HIV transmission in Haiti.

**Methods and Findings:**

A decision analytic model simulated health outcomes and costs separately for pregnant women in rural and urban areas. We compared syphilis syndromic surveillance (rural standard of care), rapid plasma reagin test with results and treatment at 1-wk follow-up (urban standard of care), and a new rapid test with immediate results and treatment. Test performance data were from a World Health Organization–Special Programme for Research and Training in Tropical Diseases field trial conducted at the GHESKIO Center Groupe Haitien d'Etude du Sarcome de Kaposi et des Infections Opportunistes in Port-au-Prince. Health outcomes were projected using historical data on prenatal syphilis treatment efficacy and included disability-adjusted life years (DALYs) of newborns, congenital syphilis cases, neonatal deaths, and stillbirths. Cost-effectiveness ratios are in US dollars/DALY from a societal perspective; annual costs are in US dollars from a payer perspective. Rapid testing with immediate treatment has a cost-effectiveness ratio of $6.83/DALY in rural settings and $9.95/DALY in urban settings. Results are sensitive to regional syphilis prevalence, rapid test sensitivity, and the return rate for follow-up visits. Integrating rapid syphilis testing into a scaled-up national HIV testing and prenatal care program would prevent 1,125 congenital syphilis cases and 1,223 stillbirths or neonatal deaths annually at a cost of $525,000.

**Conclusions:**

In Haiti, integrating a new rapid syphilis test into prenatal care and HIV testing would prevent congenital syphilis cases and stillbirths, and is cost-effective. A similar approach may be beneficial in other resource-poor countries that are scaling up prenatal HIV testing.

## Abstract 2

Analyzing data from Haiti, Bruce Schackman and colleagues report that scale-up of prenatal HIV testing programs provides a cost-effective opportunity to prevent congenital syphilis through rapid testing.

## Editors' Summary

**Background.:**

Congenital syphilis (syphilis that is passed on from a woman infected with the disease to her unborn baby) is a major preventable public health problem. Around half of all pregnancies among women infected with syphilis result in stillbirth or death of the baby shortly after birth. However, it should be possible to reduce the health burden of congenital syphilis if infections among pregnant women could be quickly and accurately diagnosed. In resource-poor countries, many syphilis infections go undiagnosed, because the tests that are normally used involve sending samples away to a laboratory for processing. This means that the diagnosis can only be confirmed, and treatment started, at the next available visit. As a result, there is a delay in starting antibiotic treatment, and some women may never receive their intended treatment at all if they cannot return for their follow-up visit. However, new tests are available that don't involve cold storage of reagents or electrical equipment, and these can be used to give an immediate result about syphilis infection even in rural or resource-poor settings. Currently, global initiatives are underway to ensure many more pregnant women are tested for HIV and to reduce the risk of HIV being passed on from a woman to her baby. These initiatives could provide an important opportunity for carrying out widespread immediate screening for syphilis during pregnancy as well. Such screening might then help reduce infant deaths substantially.

**Why Was This Study Done?:**

Field trials evaluating rapid syphilis tests have already been carried out by the World Health Organization's Special Programme for Research and Training in Tropical Diseases. One such trial, carried out in Port-au-Prince, Haiti, evaluated the success of three different rapid syphilis tests as compared to two “gold standard” tests (older tests that are generally considered reliable, but which don't give an immediate result). These researchers wanted to use data from these trials to compare costs and predicted health outcomes of including different types of syphilis screening as part of scaled-up prenatal care. Specifically, the researchers wanted to find out whether including rapid syphilis testing as part of universal prenatal care would be cost-effective and whether it would reduce the rate of stillbirths and congenital syphilis.

**What Did the Researchers Do and Find?:**

This research was based on data from the field trials previously carried out in Haiti. The data from these trials were used to create a model comparing three different strategies for screening pregnant women for syphilis infections. The three strategies were as follows: checking for the symptoms of syphilis (assumed to be the standard of care in rural areas); standard testing for antibody response to the syphilis bacterium, after which treatment can be provided at follow-up a week later (assumed to be the standard of care in urban areas); and, finally, rapid testing that gives an immediate result. For each strategy, the researchers predicted what the health outcomes would be. These outcomes are summarized in “disability-adjusted life years” (DALYs) that reflect the number of years of healthy life lost due to congenital syphilis among newborn babies, the number of stillbirths, and the number of neonatal deaths. Cost-effectiveness of each strategy was also worked out by dividing the additional costs of testing and treatment for each strategy by the number of DALYs avoided using that screening method compared to the next most expensive alternative. Under the model, urban and rural settings were looked at separately. Immediate testing was more expensive than either standard testing or checking for symptoms, but emerged as more cost-effective than standard testing in rural settings; the immediate test would cost an additional $7–$10 per disability-adjusted life year compared to the current rural or urban standard of care. The researchers predicted that if immediate syphilis testing were provided to all pregnant women in Haiti who currently have access to prenatal care, over 1,000 congenital syphilis cases would be avoided, along with over 1,000 stillbirths and neonatal deaths, at a yearly cost of $525,000.

**What Do These Findings Mean?:**

This model suggests that including rapid syphilis testing as part of current global initiatives for preventing mother-to-child transmission of HIV could substantially reduce infant deaths. The strategy is also likely to be cost-effective.

**Additional Information.:**

Please access these Web sites via the online version of this summary at http://dx.doi.org/10.1371/journal.pmed.0040183.

- MedlinePlus encyclopedia entry on congenital syphilis
- Information from the World Health Organization about congenital syphilis, including information about screening programs and new screening tests
- A report is also available from the Special Programme for Research and Training in Tropical Diseases regarding rapid syphilis tests
- AVERT, an international AIDS charity, provides information about preventing mother-to-child transmission of HIV

## Introduction

New global initiatives are financing large scale-up programs for the prevention of mother-to-child transmission of HIV in resource-poor countries [1]. However, neonates who avoid HIV infection are still at risk of dying from congenital syphilis—a disease that can largely be prevented with inexpensive penicillin treatment of the pregnant mother if correctly diagnosed [2]. An estimated 1 million pregnancies each year are adversely affected by syphilis due to maternal infection, and about half of these pregnancies end in stillbirth or neonatal death [3]. Children who survive with congenital syphilis can suffer serious long-term adverse effects such as mental retardation, deafness, and blindness [4–6]. Most women in resource-poor countries receive inadequate testing and treatment for syphilis in pregnancy, often because they must walk many hours to the testing site and fail to return for follow-up appointments. Thus, the substantial resources devoted to scaling up prenatal HIV testing and treatment services may not fully achieve the goal of reducing infant mortality unless syphilis screening becomes more widely available.

A rapid syphilis test with immediate results implemented in a field setting has the advantage of allowing women who test positive to be treated on-site at the same visit, avoiding the losses to follow-up associated with return visits and the adverse outcomes associated with delayed treatment [7,8]. The World Health Organization's Special Programme for Research and Training in Tropical Diseases has sponsored laboratory and field trials of several new commercially available rapid tests for syphilis in resource-poor countries [9]. One of the sites for these trials is the Groupe Haitien d'Etude du Sarcome de Kaposi et des Infections Opportunistes (GHESKIO), an independent non-profit center that provides clinical and laboratory services, trains health personnel, and conducts research in Port-au-Prince, Haiti. Only 68% of pregnant women in Haiti have at least one prenatal visit [10], and 66 health-care centers provide prenatal HIV testing to about 15% of all pregnant women in Haiti. With increased international support, the goal is to raise the proportion receiving prenatal care with integrated HIV testing to 85% by 2008. In urban centers, same-day syphilis screening is generally not available, and treatment is provided at follow-up visits. In rural areas, most pregnant women do not have access to syphilis screening.

We used the results of the field tests conducted at GHESKIO [9] in a decision analytic model to evaluate the cost-effectiveness, projected health outcomes, and annual cost of strategies to screen Haitian pregnant women to prevent congenital syphilis in rural and urban settings as part of scaled-up prenatal HIV testing programs.

## Methods

### Overview

We developed a decision analytic model to compare alternative strategies for adding syphilis screening to prenatal HIV testing programs. In the model, pregnant women with access to prenatal care are evaluated for syphilis at the first antenatal visit, usually at 24 wk of gestation. Depending on the syphilis screening strategy, women are informed of the result and initiate treatment either immediately or at the first follow-up visit. Treatment consists of three rounds of benzathine penicillin G given intramuscularly at approximately 1-wk intervals in accordance with US guidelines that are used in Haiti [11].

Health outcomes depend on whether the mother receives treatment, and include stillbirth, neonatal death, congenital syphilis, or an infant without congenital syphilis. To determine cost-effectiveness ratios, we calculated disability-adjusted life years (DALYs) for congenital syphilis cases [12,13]. As in a previous cost-effectiveness analysis of prenatal syphilis testing [14], we consider DALY outcomes only for the infant and not for the mother or her sexual partners, but we include DALYs averted by averting stillbirths (after taking into account the risk of stillbirth from other causes). We consider a stillbirth averted equivalent to gaining a normal disability-adjusted life expectancy at birth in Haiti. Cost-effectiveness ratios are in 2004 US dollars/DALY from a societal perspective (health system and indirect patient costs), and annual costs are in 2004 US dollars from a payer perspective. The model was programmed using DATA Pro Healthcare (TreeAge Software, http://www.treeage.com).

### Syphilis Screening Strategies

**Figure 1:**
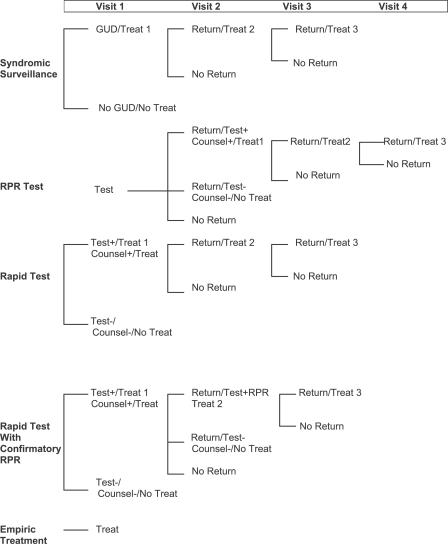
Syphilis Screening Strategies

We consider three principal syphilis screening strategies: syndromic surveillance, rapid plasma reagin (RPR) testing with results delivered at a follow-up visit approximately 1 wk later, and a rapid test with immediate results (Figure 1). Syndromic surveillance is assumed to be the standard of care in rural settings, where most patients do not have access to syphilis laboratory testing [15]. Syndromic surveillance is defined as a presumptive diagnosis of syphilis and subsequent treatment based on a finding of genital ulcer disease (GUD) at the prenatal exam. RPR testing with results delivered at a follow-up visit is assumed to be the standard of care in urban settings. Quantitative RPR testing with treatment based on titer is not done in Haiti.

Three commercially available rapid syphilis tests with immediate results were evaluated in a field test at GHESKIO (Determine Syphilis TP, Abbott Laboratories, http://www.abbott.com; SD BIOLINE Syphilis 3.0, Standard Diagnostics, http://www.standardia.com; and VISITECT Syphilis, Omega Diagnostics, http://www.omegadiagnostics.com). These tests detect antibodies specific to Treponema pallidum. Clinic nurses were trained to use each rapid test; the results of the rapid tests performed by the nurse in the field and the results of the RPR tests performed by a technician in the laboratory were compared to a gold standard of a positive reading on the Treponema pallidum haemagglutination assay (TPHA) alone; an alternative gold standard of a positive reading on both RPR and TPHA was also examined. Table 1 reports the evaluation results for pregnant women for the RPR test and one of the rapid syphilis tests compared against the TPHA gold standard; mean results for the other rapid tests generally fell within the confidence intervals used to define sensitivity analysis ranges. To eliminate the “learning curve” period associated with performing a new test, data from the first 2 wk of field use of each rapid test were excluded. The sensitivity of the rapid test on whole blood when conducted on pregnant women in field conditions was 83.3%, which is lower than previously reported when the test was conducted in the GHESKIO laboratory [9], but similar to the rapid test sensitivity when conducted at health facilities in Mozambique [16].

**Table 1:**
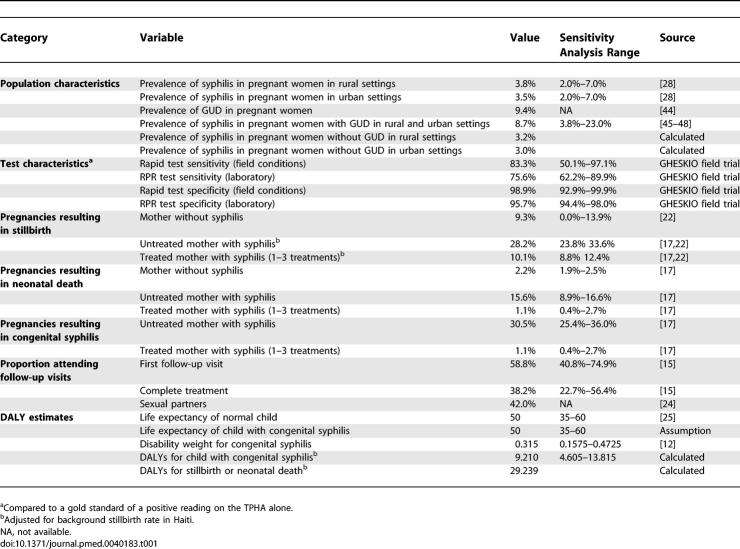
Model Input Variables for Population Characteristics, Test Performance, Treatment Outcomes, and DALYs

### Health Outcomes

US historical data on syphilis outcomes for pregnant women treated with benzathene penicillin-G offered the most appropriate estimates for these outcomes in present day Haiti (Table 1) [17]. Studies have also been conducted in Africa on the treatment outcomes of pregnant women with a positive syphilis test. However, RPR testing in Africa is of highly varying quality [18,19], there is a high prevalence of other treponemal diseases (such as yaws) that can affect RPR test performance [7], and some pregnant women may have been previously treated for syphilis, which could lead to a false positive TPHA or rapid test result. Neither competing treponemal diseases nor previous treatment for syphilis were present in the US historical study, conducted in inner-city Philadelphia in the 1940s, nor are they important factors in present-day Haiti, where yaws has been eliminated and treatment of syphilis is rare [15,20,21]. Additionally, the US historical study reported pregnancy outcomes (including stillbirths and neonatal deaths) for both treated and untreated women with syphilis and for uninfected women. Effectiveness estimates were adjusted to account for a higher background rate of stillbirths observed in uninfected women in Haiti [22].

The effectiveness of penicillin treatment in preventing congenital syphilis and stillbirths is assumed to be unaffected by failure to complete the second and third treatments [23]. Potential health benefits of second and third treatments to the pregnant woman are not considered. Return rates for pregnant women are 58.8% for the first follow-up visit and 38.2% for receiving a full course of three rounds of benzathine penicillin G [15]. All women are requested to refer their sexual partners for treatment, and we assumed that 42% of partners present for treatment [24]. DALYs for children with and without congenital syphilis are calculated based on life expectancy in Haiti and a published disability weight for congenital syphilis of 0.315 [12,13,25]. We conservatively assume that a child with congenital syphilis will have a normal life expectancy, albeit with a substantial decrement in quality of life. All DALYs are calculated using a 3% annual discount rate [26].

### Costs

Table 2 presents direct labor and material cost model inputs using market rates. Direct labor costs are derived from responses provided by the clinical staff at GHESKIO to a written survey requesting estimates of the time required during the field trial to conduct the test by a nurse (rapid test on whole blood) or by a laboratory technician (batch processing of RPR tests), and of the time required by a nurse to draw blood and to conduct pre-test and post-test counseling related to the syphilis test. When tests are performed in urban settings, we assume the average nurse labor rate of $4.91/h based on data from GHESKIO; when tests are performed in rural settings, we assume that the duties of a nurse would be performed by an aide-auxiliaire at a labor rate of $0.77/h. In the base case, the cost of the rapid test was assumed to be $1.10, the cost of the most expensive of the three tests evaluated (range $0.39–$1.10) [9]. Treatment costs are incurred by pregnant women diagnosed with syphilis and by their partners who present for treatment. Transportation, overhead, infrastructure, and training costs are not included because we assume that these costs are incurred by the integrated prenatal HIV testing program with or without syphilis testing.

**Table 2:**
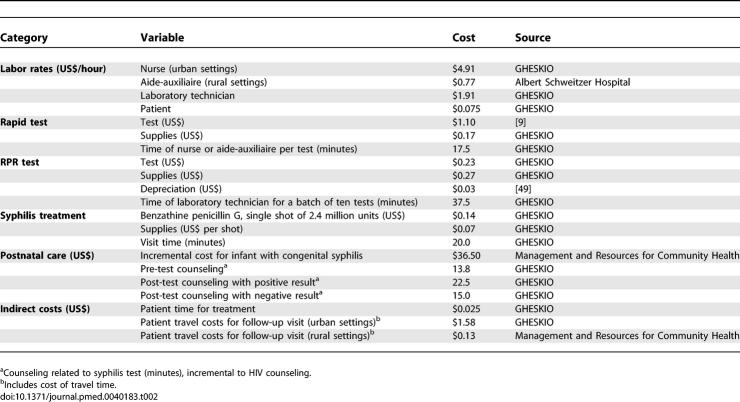
Costs Used in Model

Costs from a societal perspective also include patient travel to attend follow-up visits and patient time receiving counseling, testing, and treatment. For urban settings, we use data on the place of residence of patients attending GHESKIO clinics in 2004 and assume that 75% of patients arrive by “tap-tap” (local bus), 20% by taxi, and 5% on foot (A. St. Cyr and P. Vachon, personal communication). Transportation charges and travel times are weighted averages for patients coming from different Port-au-Prince neighborhoods. In rural settings, we assume that prenatal HIV testing sites include mobile teams that would be deployed so that women could travel on foot to a prenatal care visit (R. Berggren, personal communication). The cost of patient time is the average income self-reported by GHESKIO patients in 2004.

For children with congenital syphilis, the estimated additional cost of treatment in the first year of life compared to treatment of children without congenital syphilis is $36.50. This is the cost of three additional clinic visits ($4.50) and two hospitalizations ($24.00 for supplies and $8.00 for hospital costs). Thereafter, it is assumed that children with congenital syphilis do not receive any additional care compared to other children in Haiti because children who survive with long-term disabilities rarely have access to further diagnostic tests and treatment.

### Cost-Effectiveness Evaluation

Cost-effectiveness analyses were conducted by running the model separately for rural and urban settings, because of the differences between these settings in standards of care (syndromic surveillance versus RPR testing) and staffing patterns (nurse versus aide-auxiliaire). All cost-effectiveness ratios were calculated as the incremental cost per additional DALY averted compared with the next best strategy, after eliminating strategies because of dominance or extended dominance [26,27]. Because DALYs are measures of health losses, DALYs are averted (rather than saved) by effective interventions; therefore, more effective interventions produce fewer DALYs. A lower (more attractive) cost-effectiveness ratio minimizes the number of DALYs incurred per dollar spent [13]. One-way and two-way sensitivity analyses were conducted on model input variables. Test performance, treatment efficacy, and patient return rate sensitivity analysis ranges were based on confidence intervals derived from clinical data (Table 1). Syphilis prevalence was varied over the range observed in Haiti; life expectancy was varied by 10 y; the congenital syphilis disability weight, labor costs, and supply costs were varied by 50%; and the cost of the rapid test was varied from $0.35 to $1.50.

Two alternative testing scenarios were considered that included using the rapid test and RPR test in combination. In the first scenario, rapid testing with an RPR confirmatory test, patients with positive rapid test results commence treatment immediately, but they also receive an RPR test. At the first follow-up visit, only patients with both positive RPR test results and positive rapid test results continue treatment. In the second scenario, RPR testing with a rapid confirmatory test, patients with a positive RPR test immediately receive a rapid test at the first follow-up visit, and the rapid test must be positive in order for treatment to be initiated. We also report results for a strategy of no screening and empiric treatment for syphilis of all pregnant women presenting for antenatal care.

### Projection of National Scale-Up

We calculated the number of congenital syphilis cases, neonatal deaths, and stillbirths avoided, and the total cost of a national scale-up of rapid syphilis testing and treatment as part of integrated prenatal care and HIV testing. We ran the model separately for each of nine rural provinces (departments) and for urban settings (Port-au-Prince, Cap Haitien, and Gonaives). We used the reported prevalence of confirmed syphilis diagnosis in pregnant women and official population estimates for each location [28,29], and we multiplied the cost and outcomes per patient tested by the number of pregnant women with access to antenatal care. We assumed 100% access to antenatal care in urban areas and 64% in rural areas, based on a reported 68% national access to antenatal care [10]. Results for the provinces are aggregated in order to provide comparisons among four rural regions with populations of similar sizes (central, south, north, and west) and also include cost-effectiveness ratios from a societal perspective for each region. Costs for urban and rural regions were calculated separately, in order to derive national program costs for rapid tests, labor, supplies, and syphilis treatment.

### Patients' Consent and Ethics Committee Approval

The field trial was approved by the GHESKIO Ethics Committee and the Weill Medical College of Cornell University Institutional Review Board, and patients provided informed consent.

## Results

### Cost-Effectiveness

In rural settings, syndromic surveillance costs $0.48 per patient tested, RPR testing costs $1.43 (an incremental $0.95 versus syndromic surveillance), and rapid testing costs $2.15 (an incremental $0.72 versus RPR testing) (Table 3). RPR testing averts an incremental 0.090 DALYs per patient tested versus syndromic sureveillance, and rapid testing averts an additional incremental 0.154 DALYs versus RPR testing. The cost-effectiveness ratio for RPR testing versus syndromic surveillance is $10.64/DALY; the cost-effectiveness ratio for rapid testing versus syndromic surveillance is $6.83/DALY; and the cost-effectiveness ratio for rapid testing versus RPR testing is $4.62/DALY. Rapid testing has a lower (i.e., more attractive) cost-effectiveness ratio than RPR testing because it has greater absolute benefits in terms of DALYs averted compared to syndromic surveillance, despite its higher cost. It therefore represents a more efficient use of resources. The RPR testing strategy is eliminated because of extended dominance; it is less efficient than a mixed strategy of the rapid test and syndromic surveillance [27]. Compared to syndromic surveillance, for every 1,000 women in rural settings who receive the rapid test, 6.0 congenital syphilis cases, 6.5 stillbirths, and 4.2 neonatal deaths would be avoided.

**Table 3:**
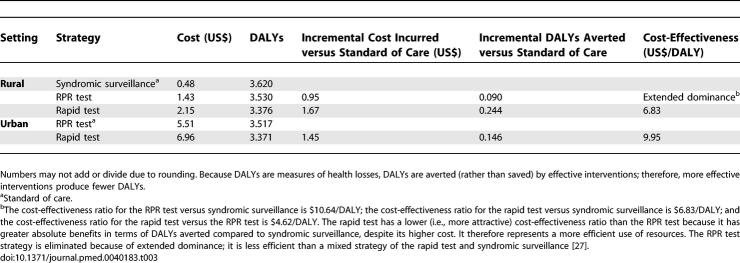
Cost-Effectiveness Results: Costs, DALYs, and Cost-Effectiveness per Maternal Patient Tested

In urban settings, RPR testing costs $5.51 per patient tested, and rapid testing costs $6.96 (an incremental $1.45) (Table 3). Rapid testing averts an incremental 0.146 DALYs compared to RPR testing, and the cost-effectiveness ratio for rapid testing is $9.95/DALY. Compared to RPR testing, for every 1,000 women in urban settings who receive the rapid test, 1.4 congenital syphilis cases, 1.0 stillbirths, and 1.1 neonatal deaths would be avoided.

In one-way sensitivity analyses, results are sensitive to the prevalence of syphilis and the sensitivity of the rapid test (Table 4). Assuming 2% prevalence of syphilis in pregnant women (versus 3.7% national prevalence), the cost-effectiveness ratios are $19.93/DALY in rural settings and $17.32/DALY in urban settings. Assuming 7% prevalence of syphilis in pregnant women, the cost-effectiveness ratios are $2.75/DALY in rural settings and $4.93/DALY in urban settings. Assuming 50% sensitivity of the rapid test (versus 83.3% in the field trial), the cost-effectiveness ratios compared to the standard of care are $15.74/DALY in rural settings and $68.98/DALY in urban settings. Assuming 97% sensitivity of the rapid test, the cost-effectiveness ratios are $5.44/DALY in rural settings and $7.35/DALY in urban settings.

**Table 4:**
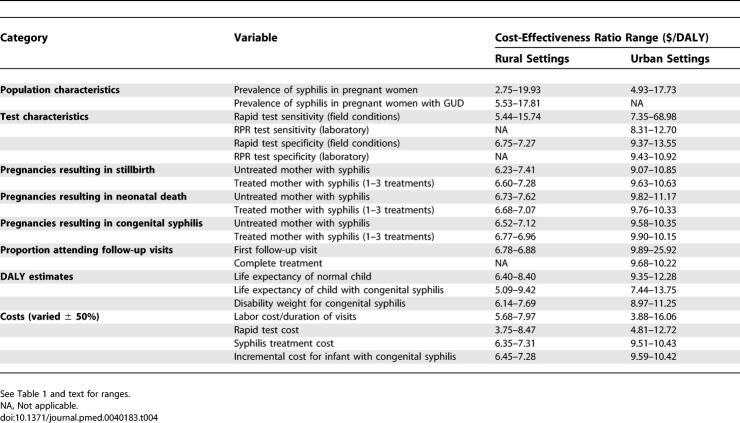
One-Way Sensitivity Analyses for Cost-Effectiveness Results Compared to the Rural and Urban Standards of Care

**Figure 2:**
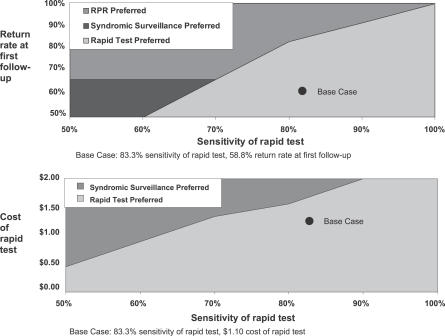
Two-Way Sensitivitity Analyses Preferred syphilis screening strategy for rural settings assuming a cost-effectiveness threshold of ≤$10/DALY (A) varying the return rate for the first follow-up visit and the sensitivity of the rapid test, and (B) varying the cost and sensitivity of the rapid test.

In two-way analyses, the impact of the sensitivity of the rapid test on cost-effectiveness results is affected by the probability that the pregnant woman returns for follow-up visits, the prevalence of syphilis, and the cost of the test. Assuming a cost-effectiveness threshold of ≤$10/DALY, rapid testing remains the preferred strategy in rural settings as long as its sensitivity is ≥75% and the return rate at the first follow-up visit is ≤75% (Figure 2A). At a price of $1.30, rapid testing remains the preferred strategy as long as its sensitivity is ≥70%, and at a price of $0.45 the rapid test remains the preferred strategy as long as its sensitivity is ≥50% (Figure 2B).

Results are more sensitive to nurse labor rates in the urban setting than in the rural setting. In the urban setting, when the nurse labor rate is varied by 50% the cost-effectiveness ratio varies from $3.42/DALY to $16.52/DALY. In the rural setting, the nurse labor rate must be 2.4 times higher than the base case in order for the cost-effectiveness ratio to exceed $10/DALY. When the sensitivity of the rapid test is low, cost-effectiveness ratios are higher (less attractive) in urban settings, where the comparator is the RPR test, than in rural settings, where the comparator is syndromic surveillance.

Results are not strongly affected by the specificity of the rapid test within the confidence interval observed in the field test nor by the partner return rate nor by test and treatment costs (Table 4). Results are not affected by the choice of gold standard; using the gold standard of a positive finding on both TPHA and RPR tests, the cost-effectiveness ratios are $5.27/DALY in rural areas and $10.00/DALY in urban areas.

Neither of the combination test scenarios is more cost-effective than the rapid test alone. In the first scenario, when RPR testing is used as a confirmatory test and treatment is discontinued if the RPR test is negative, the cost saving from eliminating unnecessary follow-up treatments for false positive rapid tests is offset by the additional cost of the RPR test unless the specificity of the rapid test is poor. In the second scenario, when the rapid test is used only as a confirmatory test on patients with a positive RPR test, the cost saving is offset by the lost opportunity to treat patients at the initial visit; this strategy always has the highest (i.e., least attractive) cost-effectiveness ratio. Empiric treatment without screening has a cost-effectiveness ratio of $2.41/DALY compared to syndromic surveillance in rural settings and $2.05/DALY compared to RPR testing in urban settings, and in sensitivity analyses it consistently has the lowest cost-effectiveness ratio. However, the feasibility and additional consequences of wide antibiotic exposure associated with this strategy were not evaluated.

### National Scale-Up

Integrating rapid syphilis screening with immediate treatment into a scaled-up national HIV testing program for the estimated 202,000 pregnant women in Haiti who currently have access to prenatal care (68% of all pregnancies) would require annually screening approximately 168,000 pregnant women in rural areas, 35,000 women in urban areas, and 85,000 sexual partners. The benefits would include avoiding 1,129 congenital syphilis cases, 786 stillbirths, and 437 neonatal deaths annually. From the payer perspective, the direct cost of this program would be $525,000 annually, of which 46% would be for rapid tests, 42% for labor, 7% for supplies, and 6% for other costs (including the costs of penicillin, partner treatment, and treatment of congenital syphilis cases). Table 5 presents the projected national scale-up results for all urban areas and the four rural regions. The cost per adverse outcome (stillbirth, neonatal death, or congenital syphilis case) avoided varies from $108 in the rural region with the highest syphilis prevalence to $218 in the rural region with the lowest prevalence; similarly, the regional cost-effectiveness ratios vary from $4.78/DALY to $10.79/DALY. Costs are highest in urban settings, because of the higher labor cost for nurses, and the incremental benefit is lower because the comparator in urban settings is RPR testing (instead of syndromic surveillance); about 90% of the adverse outcomes avoided occur in rural areas.

**Table 5:**
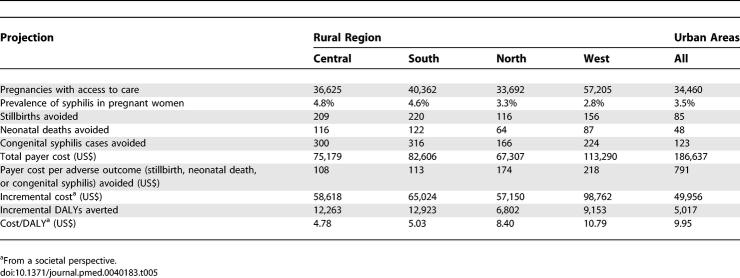
Projected Outcomes, Costs, and Incremental Cost-Effectiveness Ratios for Integrating Rapid Syphilis Screening into Prenatal HIV Testing Programs in Haiti

## Discussion

In an era when substantial resources are going towards the prevention of mother-to-child transmission of HIV, there is an opportunity to integrate these efforts with programs to prevent congenital syphilis [30,31]. New rapid syphilis tests can be included in prenatal HIV testing programs operating in settings without access to same-day laboratory results. The current study demonstrates that such a prenatal syphilis screening strategy in Haiti using new rapid tests that permit same-day diagnosis and treatment have attractive incremental cost-effectiveness ratios when compared with current practice, which relies upon syndromic management or RPR testing with treatment at a follow-up visit. We project that integrating rapid syphilis screening into a scaled-up national program to test pregnant women for HIV in Haiti would prevent over 1,100 cases of congenital syphilis and over 1,200 neonatal deaths or stillbirths annually, mostly in rural areas. The cost-effectiveness of implementing this strategy is approximately $7.00/DALY in rural settings and $10.00/DALY in urban settings. The cost to prevent an adverse outcome of syphilis in pregnancy, including a stillbirth, a neonatal death, or an infant with congenital syphilis, is $108–$218 per adverse outcome in rural settings. The results of our analysis suggest that empirically treating all pregnant women with penicillin may be more cost-effective than the current practice in settings where syphilis testing is not feasible, even though mass treatment campaigns have had mixed success in other settings [32–34]. However, our analysis did not include the potential consequences of widespread antibiotic use in the empiric treatment strategy.

The cost-effectiveness ratios of $7.00/DALY to $10.00/DALY for rapid syphilis testing fall well below two cost-effectiveness thresholds that have been recommended for health interventions in resource-poor countries: $50/DALY [35] and the average per-person gross domestic product in the country where the intervention is being implemented (in Haiti, $440) [36,37]. In addition, the cost-effectiveness ratios are similar to or lower than ratios reported for HIV interventions in sub-Saharan Africa including HIV testing in Kenya ($12.77/DALY in 1998 US dollars) and Tanzania ($17.78 in 1998 US dollars) [38], improved treatment of sexually transmitted diseases to prevent HIV infection in Tanzania ($10.33/DALY in 1993 US dollars) [39], and a 1999 study of nevirapine to prevent mother-to-child HIV transmission ($5.25–$19.18/DALY) [40].

Terris-Prestholt et al. found a similar cost-effectiveness ratio for a same-day RPR antenatal syphilis screening program in Tanzania ($10.56/DALY in 2001 US dollars) and estimated that the cost-effectiveness of this program in a variety of settings in sub-Saharan Africa is $3.97–$18.73/DALY in 2001 US dollars [14]. However, field experience in Africa has shown that there are substantial operational challenges to implementing same-day antenatal syphilis screening using RPR including adequate training, continuity of supplies, need for electricity, quality control, and supervision [18,19]. In addition, not all women are willing to wait at the testing site to obtain RPR results [18], and batch processing of RPR tests can extend waiting times. Implementing rapid syphilis screening in the context of prenatal HIV testing can address many of these challenges. Rapid syphilis tests require less training and supervision than on-site RPR tests, and results and treatment are available immediately. Logistical support and infrastructure provided by the prenatal HIV programs can be leveraged to address many of the remaining gaps [41]. Because the prenatal HIV programs already incur the cost of this support, the cost-effectiveness ratio for integrated syphilis testing is more attractive than it would be for a separate “vertical” program.

There are several limitations to our analysis. Years of life lost due to stillbirths avoided are included in our evaluation; stillbirths are not routinely included in DALY calculations and excluding them would have resulted in substantially less attractive cost-effectiveness ratios [14]. We used data from a historical US study to project treatment efficacy, which had the advantage of allowing us to project congenital syphilis cases avoided instead of using a proxy such as low birth weight. However, these data may not be representative of outcomes actually experienced in Haiti despite adjustments for the higher background stillbirth rate in Haiti. The counseling related to the syphilis test that occurred in the field trial may be more extensive than occurs in practice, but cost-effectiveness ratios in rural settings are not affected because of the low labor costs; in urban settings the cost-effectiveness ratios would be less attractive if less counseling occurs. Indirect costs for patient time and transportation were low, but may not fully reflect the personal costs associated with syphilis testing and treatment due to lost productivity and family income. Training and other start-up costs incurred by HIV testing programs were assumed to be unaffected by adding syphilis testing; however, additional start-up costs may be incurred in other settings where HIV testing is already ongoing.

The field performance of the rapid test might be improved with more intensive training and quality assurance activities, resulting in a more attractive cost-effectiveness ratio, but we did not evaluate the cost-effectiveness of such a program. RPR test performance at GHESKIO may reflect higher levels of training for its laboratory personnel compared to other urban laboratories. RPR testing in rural areas may be more expensive and less effective than our estimate because of distances between testing sites and laboratories. Because the rapid test is a trepenomal test and trepenomal antibodies can persist for life in successfully treated patients [7], the specificity of the rapid test may deteriorate in the future if treatment of syphilis becomes more widespread in Haiti. This would result in overtreatment and less attractive cost-effectiveness ratios. Using the RPR test as a confirmatory test may be cost-effective in settings where there is a high prevalence of previously treated syphilis or other endemic treponemal diseases.

Integrating rapid syphilis testing and immediate treatment into prenatal care and HIV testing in Haiti would prevent congenital syphilis cases and stillbirths, and is cost-effective. At an incremental direct cost of only $525,000 annually, the impact of adding rapid syphilis screening to the prenatal HIV testing budget appears quite modest, representing less than 1% of the $55.6 million US President's Emergency Plan for AIDS Relief budget for Haiti in fiscal year 2006 [42]. Using information on syphilis prevalence rates, test performance, and return rates for follow-up antenatal visits, projected results can help set priorities for scale-up implementation. In our analysis, these projections indicate a focus on implementing rapid syphilis testing in rural regions that have high syphilis prevalence and lack RPR testing capability.

Worldwide, effective syphilis screening could prevent more than 500,000 fetal deaths per year, primarily in sub-Saharan Africa; this result is similar to the potential benefits from preventing mother-to-child transmission of HIV [43]. We believe our findings are generalizable to HIV scale-up programs in Africa and other resource-poor settings, because in our model the cost-effectiveness ratio for rapid syphilis testing remains attractive when syphilis prevalence is varied, when rapid testing is compared to either RPR testing or syndromic surveillance, and in both rural and urban areas. Prenatal care with integrated HIV and syphilis screening will have a powerful impact on improving health outcomes in Haiti and can be a model for similar programs throughout the world.

## Abbreviations

DALY: disability-adjusted life year
GHESKIO: Groupe Haitien d'Etude du Sarcome de Kaposi et des Infections Opportunistes
GUD: genital ulcer disease
RPR: rapid plasma reagin
TPHA: *T. pallidum* hemagglutination assay
